# The Potential Role of Phytochemicals in Alzheimer’s Disease

**DOI:** 10.3390/nu17040653

**Published:** 2025-02-12

**Authors:** Ewa Baranowska-Wójcik, Dorota Gajowniczek-Ałasa, Bożena Pawlikowska-Pawlęga, Dominik Szwajgier

**Affiliations:** 1 Department of Biotechnology, Microbiology and Human Nutrition, University of Life Sciences, Skromna Street 8, 20-704 Lublin, Poland; dorota.gajowniczek@up.lublin.pl (D.G.-A.); dominik.szwajgier@up.lublin.pl (D.S.); 2 Department of Functional Anatomy and Cytobiology, Institute of Biological Sciences, Faculty of Biology and Biotechnology, Maria Curie-Sklodowska University, Akademicka 19, 20-033 Lublin, Poland; bozena.pawlikowska-pawlega@mail.umcs.pl

**Keywords:** Alzheimer’s disease, herbs, β-amyloids, AChE, tau proteins

## Abstract

Alzheimer’s disease (AD) is a neurodegenerative condition characterised by memory loss and cognitive disorders. The disease has been related to the presence of so-called senile plaques forming due to the buildup of amyloid β in the hippocampus. The AD therapies developed to date continue to prove insufficient, while long-term exposure to synthetic drugs tends to lead to serious side effects, which is why potential herbal treatments are generally preferable to conventional drug regimens and, as such, have been under considerable research scrutiny in recent years. There are a number of herbs, e.g., lavender *Ginkgo biloba*, that are already commonly employed in alleviating the symptoms of certain neurological disorders. In light of the above, the aim of the following paper is to discuss the importance of medicinal herbs, their neuroprotective properties, and their mechanisms of activity. The article presents a review of the identified therapeutic properties of phytomedicines that exhibit strong anti-Alzheimer’s disease (AD) activity.

## 1. Introduction

Alzheimer’s disease (AD) is described as a neurodegenerative disease that gradually impairs the patient’s cognitive functions and memory [1]. It is responsible for between 60 and 70% of all cases of dementia [2,3], and the number of affected patients has been growing annually, with as many as 5.3 million diagnosed AD patients in 2015 in the USA alone [4]. Currently, there are approximately 50 million patients suffering from AD worldwide, and it is estimated that by 2050, that number can reach up to 100 million [4]. The disease entails a gradual loss of memory and other cognitive functions [5,6,7], and in advanced stages, its symptoms can include hallucinations, amnesia, and disorientation, ultimately leading to the patient’s death due to malnutrition, aspiration pneumonia, dysphagia, or infection [8,9].

The exact pathogenetic mechanisms of AD are yet to be determined, but it is generally agreed that the main factors contributing to its development include excessive accumulation of insoluble amyloid β protein (Aβ) forming senile plaques in the extracellular space and on the walls of blood vessels, as well as neurofibrillary tangles (i.e., aggregates of hyperphosphorylated tau protein) [10,11,12,13]. Toxic Aβ oligomers damage mitochondria and cause neuron death and synaptic losses, all of which contribute to the neurodegenerative processes observed in AD [14,15].

There is a growing body of evidence to suggest that neuroinflammation plays a part in the pathogenesis of AD. The nuclear factor NF-κB (NF-κB) significantly influences inflammatory states, immunity, cell proliferation, and apoptosis [16]. It is considered a critical signal pathway involved in, e.g., regulating the transcription of genes (cytokine, chemokine), proinflammatory transcription factors, proinflammatory enzymes, or adhesion molecules crucial to the regulation of inflammation [17]. Astrocytes and microglia are the primary types of cells responsible for the brain’s inflammatory response [18]. The presence of aggregated protein and its abnormal folding in the brain may be related to astrocytes and microglia and leads to the release of inflammatory cytokines, which, in turn, can cause chronic inflammation of the nervous system and progression of AD [5]. Other known contributing factors include genetic factors and the toxic effects of xenobiotics (e.g., aluminium) [19]. AD also entails dyshomeostasis of metal ions as well as certain mitochondrial and oxidative disorders [1,20].

**Figure 1:**
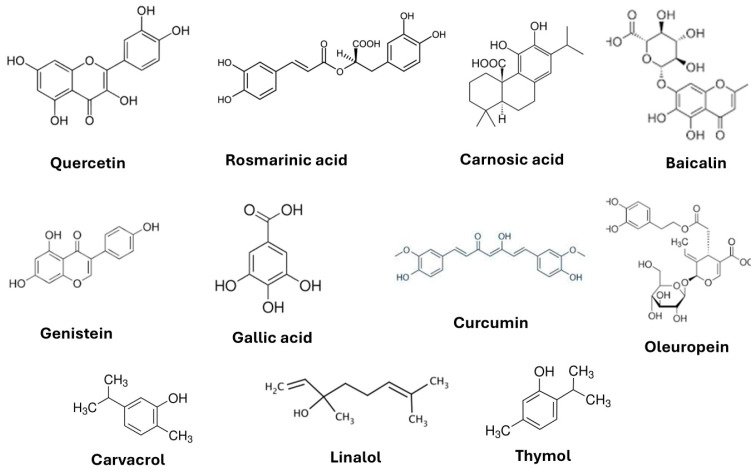
Examples of plant-derived phytochemicals for AD.

There are currently no drugs capable of effectively slowing down the progression of AD [21]. Numerous synthetic formulations, such as cholinesterase inhibitors and N-methyl-D-aspartate receptor antagonists (NMDAR) (including donepezil, rivastigmine, galantamine, and memantine) that are available on the market provide only symptomatic relief [22,23,24], as do those recently approved by the Food and Drug Administration (FDA), Aducanumab and Lecanemab, antibodies targeting Aβ [25,26,27]. This means that most therapies focus solely on symptomatic treatment, especially in the early stages of the disease [1]. Nowadays, there is an observable worldwide trend towards a more common therapeutic inclusion and recognition of herbal medicines that have long been used to alleviate certain cerebral disorders, including those inherent in AD, in some parts of the world [28,29,30]. Compared to synthetic drugs, herbal remedies are characterised by high long-term tolerance and low incidence of side effects such as sleep disorders, withdrawal syndromes, or toxic effects on other vital organs, all of which are frequent downsides of the synthetic alternatives [31,32]. Many conventional herbal and plant-based medicines contain chemical compounds with various beneficial pharmacological and biological properties [33,34] (Figure 1).

**Figure 2:**
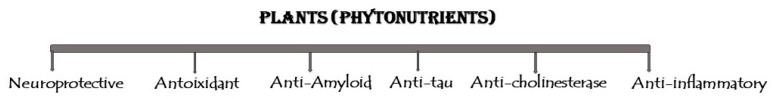
A multifaceted approach to herbal medicines in AD treatment.

Numerous plants show antioxidative and anti-inflammatory effects as well as the ability to increase acetylcholine (ACh) levels and reduce acetylcholinesterase (AChE) activity in the brain, which can significantly contribute to the treatment of AD and other neurological conditions [33,35,36,37] (Figure 2).

Phytocompounds obtained from medicinal plants contribute to cerebral chemical homeostasis by interacting with the receptors of primary inhibitory neurotransmitters [32]. Pre-clinical data suggest that regular administration of alternative preparations, such as herbal supplements, may delay and inhibit the progression of AD [38]. However, such preparations still require in-depth scientific scrutiny to determine the exact mechanisms of their neuroprotective activity [39].

## 2. Herbs in the Prevention of AD

**Figure 3:**
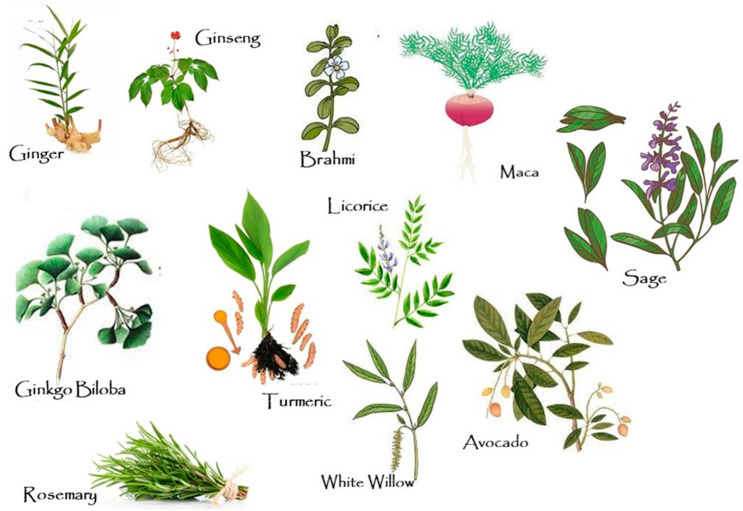
Herbs contributing to the treatment of AD.

Methods involving the use of herbal and other alternative ingredients have long been employed in traditional Indian (Ayurveda) and Chinese medicine (TCM) [40]. Unconventional treatment approaches passed down generations, discovered and perfected through long-term use and observations, continue to be employed today [33] based on a considerable body of knowledge on the beneficial properties of various plants, chemical compounds, and unique therapeutic methods [40]. TCM is rooted in over 2000 years of historical experience of Chinese physicians, while Ayurveda is perhaps the longest standing (dating back to as far as 5000 years BCE) and still most commonly employed of the six recognised medicinal systems in India (i.e., ayurveda, unani, siddha, homoeopathy, joga, and naturopathy) [33]. Certain common household spices such as ginger, cinnamon, rosemary, sage, garlic, and curcuma have also been shown to facilitate the prevention of AD (Figure 3) and other neurodegenerative disorders, owing to their content of phytochemicals [41]. For instance, rosemary was demonstrated to have neuroprotective effects facilitating the prevention of encephalitis and the formation of toxic Aβ [42]. Similarly, allicin present in garlic can inhibit the accumulation of Aβ in the human brain [43]. Studies have also confirmed the beneficial, anti-neurodegenerative properties of, e.g., ginseng, ashwagandha, *Ginkgo biloba*, *Bacopa monnieri*, and *Centella asiatica* and phytonutrients such as flavonoids, celastrol, lycopene, trehalose, sesamol, curcumin, and resveratrol [35,36,44].

Typically, a single herb or herbal mixture is used depending on whether the properties of respective plants are synergistic or whether they can modulate the activity of other compounds present in a given plant or other plant species [45,46]. Lin et al. [47] demonstrated that the therapeutic combination of two Chinese herbs, berberine and curcumin, used in a transgenic model of mice with AD, yielded a much better effect than each of the herbs alone. They observed a decrease in the production of soluble Aβ peptide (1–42) as well as a reduced inflammatory response and oxidative stress in both the cortex and hippocampus of the animals. In turn, Datta et al. [13] conducted a study on mice suffering from AD in which the animals received an ethanol extract from a single plant, *Salvia officinalis*. The authors demonstrated that the extract (300 mg/kg) significantly lowered the animals’ elevated enzyme levels and increased the tissue levels of antioxidants while also reducing the level of glutathione, as compared to the control. This evidenced the analysed herbal extract’s potential effectiveness in AD treatment.

### 2.1. Ayurveda Herbs in AD Treatment

The last scientific research has confirmed the viability of Ayurveda herbs in the prevention of AD by demonstrating the neuroprotective properties of various extracts [8]. One such plant is Asiatic pennywort (*Centella asiatica* (L.), which is capable of improving memory, purifying the blood, and lowering blood pressure. Its water extracts are commonly employed in Ayurveda to treat insomnia and facilitate the rejuvenation and rebuilding of nerve cells [48]. The side products of Asiatic pennywort, namely Asiatic acid and asiaticoside, are both strong antioxidants [49]. An in vitro study [50] demonstrated the effectiveness of Asiatic pennywort in inhibiting Aβ in mouse brains. It also acted as an antioxidant by sweeping free radicals, preventing DNA damage, and limiting lipid peroxidation. Lyle et al. [51] reported that alcohol extracts of Jatamansi root (*Nardostachys jatamansi* (D. Don) DC. (*Caprifoliaceae*)) contributed to an improvement in memory and learning functions and reduced symptoms of chronic fatigue syndrome in rats. Yet, another plant, Guggul (*Commiphora*), was shown to contain a number of effective antioxidants with potential applications in the treatment of AD, including phenols, ferulic acid, and nonphenolic aromatic acids [48]. Similarly to Asiatic pennywort, Guggul and Jatamansi show neuroprotective properties and contribute to reducing cerebral oxidative stress [10,52]. Cat’s claw (*Uncaria tomentosa*) is a tropical vine known for its immunomodulating and anti-inflammatory properties due to which it is recommended in the prevention and treatment of AD and pre-AD [46]. The conducted in vitro studies demonstrated its ability to prevent the aggregation and disaggregation of existing Aβ fibrils and tau protein tangles [53]. One of the most popular herbs in this group, *Curcuma longa*, reduces neuroinflammation in cells and lowers Aβ levels [38], regulates the cellular signal pathway, and prevents the formation of Aβ aggregates [54,55]. Kim et al. [56] demonstrated that three curcuminoids, curcumin, desmethoxycurcumin, and bisdemethoxycurcumin, protected rat pheochromocytoma PC12 and normal human endothelial cells of the umbilical vein (HUVEC) against Aβ attack (1–42). Another plant, Brahmi (*Bacopa monnieri*), also shows potential for use in the treatment of AD and other neurological disorders [57]. Its extracts contain a number of bioactive nutrients classified under various categories. In total, 20 different phytochemicals were identified in ethanol extracts of Brahmi [10]. Ashwagandha (*Withania somnifera*), also known as Indian ginseng or winter cherry [58], is one of the best-known herbs with proven effectiveness against AD. Its alkaloids facilitate improvement in cognitive functions by reducing Aβ aggregation and modulating ACh levels [59]. It was demonstrated in both in vivo and in vitro studies that ginseng shows anti-inflammatory properties related to inhibiting mediators of inflammation, such as TNF-α, NF-κB, IL-1β, and IL6 [60,61], as well as lowers the levels of the COX-2 enzyme, the primary mediator in the process of inflammation [62]. Taranalli and Cheeramkuzhy [63] evaluated ethanol extracts from the roots and aerial parts of the *Clitoria ternatea* plant (CT), also known as Shankpushpi. They demonstrated that the administration of a lower dosage (300 mg/kg) of the extract to rats with electroshock-induced amnesia resulted in an improvement in memory functions and produced an increase in levels of ACh in the brain. The root extract showed similar, albeit somewhat stronger, effects in both doses (300 and 500 mg/kg). *Rosmarinus ofiicinalis* (rosemary) is considered beneficial in cases of pathologies related to AD due to its content of cyclooxygenase-2 (COX-2) inhibitors (specifically apigenin and eugenol), which suggests anti-inflammatory properties [64]. Mukherjee et al. [65] demonstrated that asarone present in *Acorus calamus* (sweet flag) inhibits the activity of AChE, thus lowering the risk of AD onset.

The so-called Kleeb Bua Daeng (KBD) formula has long been used by local healers in Thailand in the treatment of patients suffering from deteriorating memory and insomnia. KBD is composed of three herbs: *Nelumbo nucifera* petal, *Piper nigrum* fruit, and the aerial part of *Centella asiatica* (mixed at a ratio of 1:1:1 dry weight) [66]. The authors demonstrated in their study that the KBD extract showed a dose-dependent inhibitory effect on AChE while also inhibiting aggregation of Aβ. Jyothi et al. [67] analysed the impact of *Tinospora cordifolia* on the learning capacity and memory of albino mice with alprazolam-induced amnesia. They demonstrated that the administration of alcohol extracts of *Tinospora cordifolia* significantly improved the animals’ condition, which suggests that the Indian plant may be a potential asset in the treatment of dementia and concomitant diseases such as AD [67]. Furthermore, a 30-day administration of the tropical herb *Clitoria ternatea* to newborn, young, and adult rats (100 mg/kg of root water extract) led to a significant increase in ACh levels in the hippocampus as compared to the control group animals of the same age [68]. Furthermore, methanol extract from the seeds of *Celastrus paniculatus* and its organic fractions was reported to show antioxidative and moderate anticholinesterase activity [69]. Table 1 summarises the effect of medicinal plants on anti-AD.

### 2.2. Traditional Chinese Medicine in AD Treatment

TCM uses numerous active ingredients that may facilitate the treatment of AD patients [72]. Clinical tests have demonstrated their benefits in the early prevention of the disease and their contribution to improvement in cognitive functions and brain activity in affected patients [73]. For instance, extracts from *Polygala tenuifolia* Willd. (Yuan Zhi) [74], *Polygonum multiflorum* Thunb. (He Shou Wu) [75], and *Coptis chinensis* Franch (Huang Lian) [76] were shown to alleviate cognitive deficits and aid in the repair of pathological damage in AD. Sohn et al. [77] demonstrated in their research that the oriental herbal formula called Soshiho-tang (SST) (also known as Xiaochaihu-tang in China and Sho-saiko-to in Japan) inhibited the activation of AChE and aggregation of Aβ in vivo in a mouse model of AD. Jin et al. [78] reported that Baicalin (BAI) (a natural flavonoid isolated from *Scutellaria baicalensis* Georgi (Huang Qin)) can relieve neuroinflammation caused by the microglia in the brains of AD mice by suppressing the activation of inflammasomes NLRP3 and the TLR4/NF-κB signal pathway. *Sesamum indicum* L. (Zhi Ma) is rich in sesame oil (SO), and it was shown by Mohamed et al. [79] that SO significantly improved the learning capacity and alleviated memory loss in rats receiving AlCl_3_ (100 mg/kg) alone or with SO. It was also shown that the administration of AlCl_3_ led to histopathological changes, a reduction in cerebral oxidative stress, and an increased expression of tumour necrosis factor-alpha (TNF-α) and interleukin-1 beta (IL-1β). Cuya et al. [80] reported that the compounds present in ginger root extract ((E)-1,7-bis(4-hydroxy-3-methoxyphenyl)hept-4-en-3-on and 1-(3,4-dihydroxy-5-methoxyphenyl)-7-(4-hydroxy-3-ethoxyphenyl) heptane-3,5-diyl diacetate) could inhibit AChE in humans [1,80]. Geniposide, an iridoid compound present in gardenia fruit (*Gardenia jasminoides* ElLis), protects neurons against the neurotoxicity induced by Aβ by activating the glucagon-like peptide-1 receptor (GLP-1R) [81]. The extract from *Hypericum perforatum* (HPE) improved the cognitive function in rats with AD. To induce behavioural, biochemical, and neurochemical symptoms similar to AD, the rats received AlCl_3_. After 31 days and until the end of the experiment (for 60 days), the animals also received the HPE extract [82]. The authors observed reduced levels of noradrenaline and dopamine as well as weaker activity of AChE, decreased glutamic acid levels, reduced Aβ aggregation, and neurotransmitter modulation in the rats. Clinical tests conducted on AD patients confirmed the beneficial effects of another traditional Chinese herb, *Ginkgo biloba*, in terms of improved cognitive function, particularly in the early stages of the disease [83,84]. Yancheva et al. [85] demonstrated that *G. biloba* can stimulate neurotransmitter activity, contributing to improved learning capacity and memory in AD, as well as normalising the ACh receptors in the hippocampus. *G. biloba* was also reported to show a strong ability to inhibit AChE [86], while in vitro studies demonstrated that the plant’s extract can prevent the oligomerisation of Aβ and the formation of fibrils [87]. It also has neuroprotective effects, as it regulates the phosphorylation of tau protein [88]. Other authors observed that water extracts of lavender (*Lavandula angustifolia*) are capable of sweeping free radicals and show strong antioxidative activity. Moreover, in AD rats that were given lavender dosed at 100 and 200 mg/kg, a clear reduction in the sizes of Aβ aggregates was reported [89]. One of the most popular plants, *Melissa officinalis,* also known simply as Melissa, was attributed with anti-inflammatory, antidepressant, and antianxiety properties [24,90,91], as well as the ability to alleviate neuronal excitability and improve cognitive functions in AD patients [92]. It also shows neuroprotective activity by reducing induced Aβ [93]. Other authors suggested that the Chinese herbal formula known as Tiaoxin may prove useful in the treatment of early stages of AD [94]. They administered the preparation to mice with AD and observed a significant decrease in the accumulation of Aβ plaques in the cerebral cortex and the hippocampus, the concentration of Ab1-42 in blood serum, and the expression of miR-34a in APP/PS1 mice [95].

It was also demonstrated that essential oils extracted from plants from the mint family can improve cognitive functions in patients suffering from AD [20]. The essential oil from Spanish sage (*Salvia lavandulaefolia*) and its respective monoterpenoids are known to inhibit AChE both in vitro and in vivo [95]. The monoterpenoids present in thyme (*Thymus vulgaris*) essential oil, such as carvacrol, thymol, and linalool, inhibit AChE in vitro [96]. Moreover, essential oils obtained from marjoram, camomile, lavender, and rosemary were shown to significantly reduce agitation in AD patients [97]. Eskandari-Roozbahani et al. [98] conducted an experiment using *Zataria multiflora* essential oil in a rat AD model and reported reduced AChE activity in the rats’ hippocampi and increased levels of BDNF (Brain-Derived Neurotrophic Factor) without changes in terms of the antioxidative status. Table 2 summarises the effects of medicinal plants on anti-AD.

## 3. Conclusions

In recent years, there has been an increase in the number of cases of neurodegenerative diseases, which are closely linked to an ageing population. AD remains incurable and is the leading cause of dementia among the elderly. Key biomarkers in this context include excessive accumulation of Aβ42 and phosphorylated tau protein. One therapeutic approach is the elimination of these deposits by inhibiting Aβ aggregation. Intensive research conducted worldwide on its causes offers hope for more effective therapies for patients with AD; however, many currently available diagnostic and therapeutic methods show limited or no clinical effectiveness, primarily due to their restricted action. The medications used in AD therapy mainly consist of AChE inhibitors and NMDAR inhibitors (such as rivastigmine, galantamine, donepezil, and memantine). However, their effects are limited to alleviating symptoms and slowing disease progression. Contemporary research focuses on developing effective diagnostic methods and searching for new drugs. There is significant potential in discovering drugs based on plant extracts. Plants such as *Ginkgo biloba*, ashwagandha, and ginseng have beneficial effects on brain health and the nervous system, which may lead to improvements in memory and concentration, and a reduction in dementia symptoms. Currently used medications for AD are administered in the later stages of the disease when there is no possibility of reversing neuronal damage. Their action involves alleviating key symptoms that affect patients’ cognitive abilities and daily functioning. However, treatment effects are time-limited, and the need to increase doses leads to side effects such as nausea and vomiting. Various plant components and their extracts may have therapeutic effects on symptoms related to AD with minimal side effects due to their anti-inflammatory and antioxidant properties. This article aims to demonstrate the role of plant extracts and their neuroprotective properties as alternative intervention methods in treating AD. These extracts contain significant metabolites that influence the mechanisms associated with AD, offering a range of beneficial effects such as antioxidant and anti-inflammatory actions as well as improved cognitive functions. Additionally, these extracts may support AD therapy by stimulating the degradation of Aβ. Many plants possess antioxidant and anti-inflammatory properties while also affecting improvement in the ACh levels or limiting AChE in the brain, which may aid in treating AD and other neurological disorders. We believe that herbal treatment can act protectively and have a therapeutic impact on the symptoms associated with AD. In fact, a substantial amount of evidence indicates that AD is consistently accompanied by increased oxidative stress in the brain cells caused by the elevated production of free radicals, reduced levels of polyunsaturated fatty acids, increased oxidation of proteins and DNA, elevated lipid peroxidation, and aggregation and accumulation of Aβ, leading to oxidative stress. In terms of neuroprotection, substances such as polyphenols and plant-derived triterpenes may have anti-inflammatory and antioxidant effects, which are significant in the therapy of neurodegenerative diseases. Regular use of these plants may support cognitive functions and overall quality of life for individuals struggling with diseases such as AD. In summary, there is an urgent need to develop specific strategies aimed at preventing, halting, or slowing disease progression. Further research is also necessary to assess the safety and toxicity of such therapies. Clinical studies regarding the treatment methods in AD therapy are still relatively underdeveloped compared to other conditions. We believe that introducing drugs based on plant extracts could provide real hope for patients with AD.

## Figures and Tables

**Table 1:** Potential anti-AD effects of medicinal plants.

Plant/Herbal Formula	Main Active Compounds	Dose/Exposure Time	Model	Neuroprotective Effects	Reference
Asiatic pennywort (*Centella asiatica* L.)	Phenols, Flavonoids	2.5 or 5.0 g/kg/day, for 2 or 8 months	Mouse	- Scavenging free radicals - Reducing lipid peroxidation - Protecting against DNA damage - Impact the Aβ cascade	[50,55]
Jatamansi (*Nardostachys jatamansi*)	Flavonoids, Polyphenols, Glycoside, Thankuniside, Triterpene	200 and 500 mg/kg for 31 days	Rats	- Antioxidant effect - Reduce locomotor activity of the stressed group	[51]
*Curcuma longa*	Curcumin	7.5 mg/kg/day for 7 days	Mice	- Clears and reduces existing plaques - Curcumin facilitated the reversal of neurotoxicity by influencing Aβ accumulation in a mouse AD model.	[70]
Ashwagandha (*Withania somnifera*)	Withanolides, Alkaloids	200 and 500 mg/kg for 30 days	Mice	- Antioxidant, anti-inflammatory, and anti-fibrilogenic activities - Reversed behavioural deficits, plaque pathology, accumulation of Aβ, and oligomers in the brains of APP/PS1 AD transgenic mice	[44,59]
Rosemary (*Rosmarinus officinalis*)	Carnosic acid, Rosmarinic acid, Carnosol	10, 20, 100, 250, 500, or 1000 μg/cm^2^ of RE or 2, 10 or 20 μg/cm^2^ of CA, 24 h	Mice	- Anti-inflammatory properties - Reduced the expression of IL-1β and TNF-α	[44,64]
Kleeb Bua Daeng (KBD)	Phenols, Flavonoids	100 and 500 mg/kg/day for 7 days	Mice	- Inhibitory effect on AChE - Antioxidant activity - KBD could improve scopolamine-induced memory deficit in mice	[66]
*Tinospora cordifolia*	Alkaloids (Choline), Phenolics	140 mg/kg and 280 mg/kg for 14 days	Mice	- Alcoholic extract has enhanced cognition mouse	[67]
*Clitoria ternatea* (*C. ternatea*)	Alkaloids, Sapoins, Flavonoids, Coumarins Lignans	100 mg/kg for 30 days	Rats	- Significantly increased ACh	[68]
		100, 200 and 300 mg/kg for 28 days	Rats	- Reduced the extent of neuron damage in the CA1 hippocampus region - The higher dosage triggered significant inhibition of AChE activity in the frontal cortex and hippocampus of the rats	[71]

**Table 2:** Potential anti-AD effects of medicinal plants.

Plant/Herbal Formula	Main Active Compounds	Dose/Exposure Time	Model	Neuroprotective Effects	Reference
Soshiho-tang (SST)	Baicalin, Baicalein, Wogonin, Liquiritin, Glycyrrhizin	500, 1000, or 2000 mg/kg/day for 20 days	Mice	- Improves memory impairments in AD-like mice by protecting neurons and inhibiting inflammation in the hippocampus and cortex - Ameliorated amount of Aβ in brain	[77]
*Scutellaria baicalensis*	Flavonoids (Baicalin)	100 mg/kg for 33 days	Mice	- Treatment attenuated spatial memory dysfunction - Decreased the number of activated microglia and proinflammatory cytokines - Inhibited microglia-induced neuroinflammation	[78]
*Sesamum indicum* L.	Sesame oil (sesamin)	1 mL/kg or 2 mL/kg for 6 weeks	Mice	- The use of SO reduced the elevated overexpression of AChE and Aβ - Reduced oxidative stress	[79]
*Hypericum perforatum*	Catechin, Quercetin, Resveratrol, Curcumin, Isoflavones	300 mg/kg for 90 days	Rats	- Increase in AChE activity - Increased glutamic acid level - Decreased noradrenaline and dopamine levels - Reversed AlCl 3-induced hippocampal pathology (including Aβ)	[82,99]
*Ginkgo biloba*	Flavonoids (Quercetin, Kaempferol, Isorhamnetin), terpenoids	240 mg/day for 22 weeks	Outpatients	- Antioxidant and anti-inflammatory effects - stimulate neurotransmitter activity - Improved learning capacity and memory in AD	[44,49,85]
Lavender (*Lavandula angustifolia)*	Linalool, Linalyl Acetate	6.25, 12.5, 25, 50, and 100 μg/mL for 24 h	Human hepatoma G2 (HepG2)	- Inhibits the formation of Aβ aggregates - Diminished Aβ fibril formation - Free radical scavenging	[57]
Tiaoxin	Flavonoids Kaempferol Apigenin	0.057 g/day for 12 weeks	Mice	- Treatment significantly - Reduced memory impairment - Reduced Aβ plaque accumulation - Reduced miR-34a expression	[49,94]
*Zataria multiflora*	Carvacrol, Thymol, p-Cymene	100 μL/kg/day for 20 days	Rats	- Reduced hippocampal AchE activity - Better outcomes in Morris Water Maze (MWM) test	[98]

## References

[1] Noori T., Dehpour A.R., Sureda A., Sobarzo-Sanchez E., Shirooie S. (2021). Role of natural products for the treatment of Alzheimer’s disease. Eur. J. Pharmacol..

[2] Leng F., Edison P. (2021). Neuroinflammation and microglial activation in Alzheimer disease: Where do we go from here?. Nat. Rev. Neurol..

[3] Zeng Q., Siu W., Li L., Jin Y., Linag S., Cao M., Ma M., Wu Z. (2019). Autophagy in Alzheimer’s disease and promising modulatory effects of herbal medicine. Exp. Gerontol..

[4] Cai Z., Wang C., Yang W. (2016). Role of berberine in Alzheimer’s disease. Neuropsychiatr. Dis. Treat..

[5] Ding M.R., Qu Y.J., Hu B., An H.M. (2022). Signal pathways in the treatment of Alzheimer’s disease with traditional Chinese medicine. Biomed. Pharmacol..

[6] Dai Z., Hu T., Wei J., Wang X., Cai C., Gu Y., Hu Y., Wang W., Wu Q., Fang J. (2024). Network-based identification and mechanism exploration of active ingredients against Alzheimer’s disease via targeting endoplasmic reticulum stress from traditional chinese medicine. Comput. Struct. Biotechnol. J..

[7] Alexandre-Silva V., Pereira G.C., Ribeiro A.M. (2024). Therapeutic approaches using natural substances on the streptozotocin-induced animal model of sporadic Alzheimer’s disease: A systematic review. Adv. Tradit. Med..

[8] Kalia M. (2003). Dysphagia and aspiration pneumonia in patients with Alzheimer’s disease. Metabolism.

[9] Pandey S.N., Rangra N.K., Singh S., Arora S., Gupta V. (2021). Evolving role of natural products from traditional medicinal herbs in the treatment of Alzheimer’s disease. ACS Chem. Neurosci..

[10] Dubey T., Chinnathambi S. (2019). Brahmi (*Bacopa monnieri*): An ayurvedic herb against the Alzheimer’s disease. Arch. Biochem. Biophys..

[11] Baranowska-Wójcik E., Szwajgier D. (2020). Alzheimer’s disease: Review of current nanotechnological therapeutic strategies. Expert Rev. Neurother..

[12] Khan S., Barve K.H., Kumar M.S. (2020). Recent advancements in pathogenesis, diagnostics and treatment of Alzheimer’s disease. Curr. Neuropharmacol..

[13] Datta S., Patil S. (2020). Evaluation of Traditional Herb Extract *Salvia officinalis* in Treatment of Alzheimers Disease. Pharmacogn. J..

[14] Reiss A.B., Arain H.A., Stecker M.M., Siegart N.M., Kasselman L.J. (2018). Amyloid toxicity in Alzheimer’s disease. Rev. Neurosci..

[15] Arnsten A.F.T., Datta D., Tredici K.D., Braak H. (2021). Hypothesis: Tau pathology is an initiating factor in sporadic Alzheimer’s disease. Alzheimer’s Dement..

[16] Giridharan S., Srinivasan M. (2018). Mechanisms of NF-κB p65 and strategies for therapeutic manipulation. J. Inflamm. Res..

[17] Shih R.H., Wang C.Y., Yang C.M. (2015). NF-kappaB signaling pathways in neurological inflammation: A mini review. Front. Mol. Neurosci..

[18] Woo J.H., Lee J.H., Kim H., Park S.J., Joe E.H., Jou I. (2015). Control of inflammatory responses: A new paradigm for the treatment of chronic neuronal diseases. Exp. Neurobiol..

[19] Peric A., Annaert W. (2015). Early etiology of Alzheimer’s disease: Tipping the balance toward autophagy or endosomal dysfunction?. Acta Neuropathol..

[20] Agatonovic-Kustrin S., Kustrin E., Morton D.W. (2019). Essential oils and functional herbs for healthy aging. Neural Regen. Res..

[21] Hodson R. (2018). Alzheimer’s disease. Nature.

[22] Fink H.A., Linskens E.J., MacDonald R., Silverman P.C., McCarten J.R., Talley K.M.C., Forte M.L., Desai P.J., Nelson V.A., Miller M.A. (2020). Benefits and harms of prescription drugs and supplements for treatment of clinical Alzheimer-type dementia: A systematic review and meta-analysis. Ann. Intern. Med..

[23] You J., Li C., Chen W. (2020). A network pharmacology-based study on Alzheimer disease prevention and treatment of Qiong Yu Gao. BioData Min..

[24] Soheili M., Karimian M., Hamidi G., Salami M. (2021). Alzheimer’s disease treatment: The share of herbal medicines. Iran. J. Basic Med. Sci..

[25] Ma Y., Liu S., Zhou Q., Li Z., Zhang Z., Yu B. (2024). Approved drugs and natural products at clinical stages for treating Alzheimer’s disease. Chin. J. Nat. Med..

[26] Huang L.K., Kuan Y.C., Lin H.W., Hu C.J. (2023). Clinical trials of new drugs for Alzheimer disease: A 2020-2023 update. J. Biomed. Sci..

[27] Conti Filho C.E., Loss L.B., Marcolongo-Pereira C., Rossoni Junior J.V., Barcelos R.M., Chiarelli-Neto O., Silva B.S.d., Passamani Ambrosio R., Castro F.C.d.A.Q., Teixeira S.F. (2023). Advances in Alzheimer’s disease’s pharmacological treatment. Front. Pharmacol..

[28] Wu J., Wang Y., Zhang Z., Yu B. (2014). Herbal medicine in the treatment of Alzheimer’s disease. Chin. J. Integr. Med..

[29] Soheili M., Khalaji F., Mirhashemi M., Salami M. (2018). The effect of essential oil of Lavandula angustifolia on amyloid beta polymerization: An in vitro study. Iran. J. Chem. Chem Eng..

[30] Oseni O.A., Okoh O.S., Kayode A.A. (2020). Acetylcholinesterase inhibition and antioxidant potentials of some Nigerian medicinal plants for the treatment of alzheimer disease and other related complications. J. Nat. Prod. Res..

[31] Yadav A., Jangra M., Kr P. (2014). Herbal and synthetic approaches for the treatment of epilepsy. Int. J. Nutri..

[32] Lalotra S., Vaghela J.S. (2019). Scientific reports of medicinal plants used for the prevention and treatment of neurodegenerative diseases. Pharm. Biosci. J..

[33] Sharma R., Kuca K., Nepovimova E., Kabra A., Rao M.M., Prajapati P.K. (2019). Traditional Ayurvedic and herbal remedies for Alzheimer’s disease: From bench to bedside. Expert Rev. Neurother..

[34] Zhao Z., Yuan Y., Li S., Wang X., Yang X. (2024). Natural compounds from herbs and nutraceuticals as glycogen synthase kinase-3β inhibitors in Alzheimer’s disease treatment. CNS Neurosci. Ther..

[35] Ratheesh G., Tian L., Venugopal J.R., Ezhilarasu H., Sadiq A., Fan T.P., Ramakrishna S. (2017). Role of medicinal plants in neurodegenerative diseases. Biomanuf. Rev..

[36] Mehla J., Gupta P., Pahuja M., Diwan D., Diksha D. (2020). Indian Medicinal Herbs and Formulations for Alzheimer’s Disease, from Traditional Knowledge to Scientific Assessment. Brain Sci..

[37] Rajamanickam G., Manju S.L. (2021). A review on phyto-nanotechnology for therapy of alzheimer’s disease. Indian J. Biochem. Biophys..

[38] Sivaraman D., Anbu N., Kabilan N. (2019). Review on current treatment strategy in Alzheimer’s disease and role of herbs in treating neurological disorders. Int. J. Trans. Res. Ind. Med..

[39] Singh A., Agarwal S., Singh S. (2019). Age related neurodegenerative Alzheimer’s disease: Usage of traditional herbs in therapeutics. Neurosci. Lett..

[40] Patwardhan B.A. (2020). The designer medicine, review of ethnopharmacology and Bioprospecting Research. Indian Drugs.

[41] Hügel H.M. (2015). Brain food for Alzheimer-free ageing: Focus on herbal medicines. Adv. Exp. Med. Biol..

[42] Habtemariam S. (2016). The therapeutic potential of rosemary (*Rosmarinus officinalis*) diterpenes for Alzheimer’s disease. Evid.-Based Complement. Alternat. Med..

[43] Gupta V.B., Indi S.S., Rao K.S.J. (2009). Garlic extract exhibits antiamyloidogenic activity on amyloid-beta fibrillogenesis: Relevance to Alzheimer’s disease. Phytother. Res..

[44] Bordoloi S., Pathak K., Devi M., Saikia R., Das J., Kashyap V.H., Das D., Ahmad M.Z., Abdel-Wahab B.A. (2024). Some promising medicinal plants used in Alzheimer’s disease: An ethnopharmacological perspective. Discov. Appl. Sci..

[45] Rasoanaivo P., Wright C.W., Willcox M.L., Gilbert B. (2011). Whole plant extracts versus single compounds for the treatment of malaria: Synergy and positive interactions. Malaria J..

[46] Gregory J., Vengalasetti Y.V., Bredesen D.E., Rao R.V. (2021). Neuroprotective herbs for the management of Alzheimer’s disease. Biomolecules.

[47] Lin L., Li C., Zhang D., Yuan M., Chen C.H., Li M. (2020). Synergic effects of berberine and curcumin on improving cognitive function in an Alzheimer’s disease mouse model. Neurochem. Res..

[48] Singh A.K., Rai S.N., Maurya A., Mishra G., Awasthi R., Shakya A., Chellappan D.K., Dua K., Vamanu E., Chaudhary S.K. (2021). Therapeutic potential of phytoconstituents in management of Alzheimer’s disease. Evid.-Based Complement. Alternat. Med..

[49] Kushwah S., Maurya N.S., Kushwaha S., Scotti L., Chawade A., Mani A. (2023). Herbal therapeutics for alzheimer’s disease: Ancient Indian medicine system from the modern viewpoint. Curr. Neuropharmacol..

[50] Dhanasekaran M., Holcomb L.A., Hitt A.R., Tharakan B., Porter J.W., Young K.A., Manyam B.V. (2009). *Centella asiatica* extract selectively decreases amyloid β levels in hippocampus of alzheimer’s disease animal model. Phytotherapy Res..

[51] Lyle N., Gomes A., Sur T., Munshi S., Paul S., Chatterjee S., Bhattacharyya D. (2009). The role of antioxidant properties of *Nardostachys jatamansi* in alleviation of the symptoms of the chronic fatigue syndrome. Behav. Brain Res..

[52] Rao R.V., Descamps O., John V., Bredesen D.E. (2012). Ayurvedic medicinal plants for Alzheimer’s disease: A review. Alzheimers Res. Ther..

[53] Snow A.D., Castillo G.M., Nguyen B.P., Choi P.Y., Cummings J.A., Cam J., Hu Q., Lake T., Pan W., Kastin A.J. (2019). The Amazon rain forest plant *Uncaria tomentosa* (cat’s claw) and its specific proanthocyanidin constituents are potent inhibitors and reducers of both brain plaques and tangles. Scient Rep..

[54] Sahiner M., Yilmaz A.S., Gungor B., Sahiner N. (2023). A review on phyto-therapeutic approaches in alzheimer’s disease. J. Funct. Biomater..

[55] Sharma H., Chandra P. (2024). Effects of natural remedies on memory loss and Alzheimer’s disease. Afr. J. Biol. Sci..

[56] Kim D.S., Park S.Y., Kim J.Y. (2001). Curcuminoids from *Curcuma longa* L. (Zingiberaceae) that protect PC12 rat pheochromocytoma and normal human umbilical vein endothelial cells from βA (1–42) insult. Neurosci. Lett..

[57] Pramanik R., Dey A., Chakrabarty A.K., Banerjee D., Narwaria A., Sharma S., Rai R.K., Kati C.K., Dubay S.K. (2024). Diabetes Mellitus and Alzheimer’s Disease: Understanding Disease Mechanisms, their Correlation, and Promising Dual Activity of Selected Herbs. J. Ethnopharmacol..

[58] Menghani Y.R., Bhattad D.M., Chandak K.K., Taksande J.R. (2021). A Review: Pharmacological and herbal remedies in The Management of Neurodegenerative disorder (Alzheimer’s). Int J. Pharamcogn..

[59] Sehgal N., Gupta A., Valli R.K., Joshi S.D., Mills J.T., Hamel E., Khanna P., Jain S.C.H., Thakur S.S., Ravindranath V. (2012). Withania somnifera reverses Alzheimer’s disease pathology by enhancing low-density lipoprotein receptor-related protein in liver. Proc. Natl. Acad. Sci. USA.

[60] Song S.B., Tung N.H., Quang T.H., Ngan N.T.T., Kim K.E., Kim Y.H. (2012). Inhibition of TNF-α-mediated NF-κB Transcriptional Activity in HepG2 Cells by Dammarane-type Saponins from Panax ginseng Leaves. J. Ginseng. Res..

[61] Ahn S., Singh P., Castro-Aceituno V., Simu S.Y., Kim Y.J., Mathiyalagan R., Yang D.C. (2017). Gold nanoparticles synthesized using Panax ginseng leaves suppress inflammatory-mediators production via blockade of NF-κB activation in macrophages. Artif. Cells Nanomed. Biotechnol..

[62] Lee S.M. (2014). Anti-inflammatory Effects of Ginsenosides Rg5, Rz1, and Rk1: Inhibition of TNF-α-induced NF-κB, COX-2, and iNOS transcriptional expression. Phytother. Res..

[63] Taranalli A.D., Cheeramkuzhy T.C. (2020). Influence of *Clitoria ternatea* extracts on memory and central cholinergic activity in rats. Pharm. Biol..

[64] Mengoni E.S., Vichera G., Rigano L.A., Rodriguez-Puebla M.L., Galliano S.R., Cafferata E.E., Pivetta O.H., Moreno S., Vojnov A.A. (2011). Suppression of COX-2, IL-1β and TNF-α expression and leukocyte infiltration in inflamed skin by bioactive compounds from *Rosmarinus officinalis* L.. Fitoterapia.

[65] Mukherjee P.K., Kumar V., Mal M., Houghton P.J. (2007). In vitro acetylcholinesterase inhibitory activity of the essential oil from *Acorus calamus* and its main constituents. Planta Med..

[66] Chheng C., Waiwut P., Plekratoke K., Chulikhit Y., Daodee S., Manthakantirat O., Pitiporn S., Musigavong N., Kwankhao P., Boonyarat C. (2020). Multitarget activities of Kleeb Bua Daeng, a Thai traditional herbal formula, against Alzheimer’s disease. Pharm..

[67] Jyothi C.H., Shashikala G., Vidya H.K., Shashikala G.H. (2016). Evaluation of effect of alcoholic extract of *Tinospora cordifolia* on learning and memory in alprazolam induced amnesia in albino mice. Int. J. Basic Clin. Pharm..

[68] Rai K.S., Murthy K.D., Karanth K.S., Nalini K., Rao M.S., Srinivasan K.K. (2002). Clitoria ternatea root extract enhances acetylcholine content in rat hippocampus. Fitoterapia..

[69] Alama B., Haque E. (2011). Anti-alzheimer and antioxidant activity of *Celastrus paniculatus* seed. Iran. J. Pharm. Sci..

[70] Garcia-Alloza M., Borrelli L.A., Rozkalne A., Hyman B.T., Bacskai B.J. (2007). Curcumin labels amyloid pathology in vivo, disrupts existing plaques, and partially restores distorted neurites in an Alzheimer mouse model. J. Neurochem..

[71] Damodaran T., Cheah P.S., Murugaiyah V., Hassan Z. (2020). The nootropic and anticholinesterase activities of *Clitoria ternatea* Linn. root extract: Potential treatment for cognitive decline. Neurochem Int..

[72] Zhang Y., Lin C., Zhang L., Cui Y., Gu Y., Guo J., Wu D., Li Q., Song W. (2015). Cognitive improvement during treatment for mild Alzheimer’s disease with a Chinese herbal formula: A randomized controlled trial. PLoS ONE.

[73] Zhang J., Yang C., Wei D., Li H., Leung E.L.H., Deng Q., Liu Z., Fan X.X., Zhang Z. (2019). Long-term efficacy of Chinese medicine Bushen Capsule on cognition and brain activity in patients with amnestic mild cognitive impairment. Pharmacol. Res..

[74] Park H., Kang S., Nam E., Suh Y.H., Chang K.A. (2019). The protective effects of PSM-04 against beta amyloid-induced neurotoxicity in primary cortical neurons and an animal model of Alzheimer’s disease. Front. Pharmacol..

[75] Ning F., Chen L., Chen L., Liu X., Zhu Y., Hu J., Xie G., Xiu J., Shi K., Lan Z. (2021). Combination of polygoni multiflori radix praeparata and acori tatarinowii rhizoma alleviates learning and memory impairment in scopolamine-treated mice by regulating synaptic-related proteins. Front. Pharmacol..

[76] Chen Y., Chen Y., Liang Y., Chen H., Ji X., Huang M. (2020). Berberine mitigates cognitive decline in an Alzheimer’s Disease Mouse Model by targeting both tau hyperphosphorylation and autophagic clearance. Biomed. Pharmacol..

[77] Sohn E., Kim Y.J., Jeong S.J. (2021). Korean traditional herbal formula Soshiho-tang attenuates memory impairment and neuronal damage in mice with amyloid-beta-induced Alzheimer’s disease. Integr. Med. Res..

[78] Jin X., Liu M.Y., Zhang D.F., Zhong X., Du K., Qian P., Yao W.F., Gao H., Wei M.J. (2019). Baicalin mitigates cognitive impairment and protects neurons from microglia-mediated neuroinflammation via suppressing NLRP 3 inflammasomes and TLR 4/NF-κB signaling pathway. CNS Neurosci. Ther..

[79] Mohamed E.A., Ahmed H.I., Zaky H.O., Badr A.M. (2021). Sesame oil mitigates memory impairment, oxidative stress, and neurodegeneration in a rat model of Alzheimer’s disease. A pivotal role of NF-κB/p38MAPK/BDNF/PPAR-γ pathways. J. Ethnopharmacol..

[80] Cuya T., Baptista L., Costa França T.C. (2018). A molecular dynamics study of components of the ginger (*Zingiber officinale*) extract inside human acetylcholinesterase: Implications for Alzheimer disease. J. Biomol. Struct. Dyn..

[81] Liu J., Yin F., Guo L., Gu G., Gao R., Peng B., Wang Y., Li A., Guo J., Xu X. (2015). Molecular Mechanisms of Geniposide and Genipin Against Alzheimer’s Disease. Bioact. Nutraceuticals Diet. Suppl. Neurol. Brain Dis..

[82] Cao Z., Wang F., Xiu C., Zhang J., Li Y. (2017). Hypericum perforatum extract attenuates behavioral, biochemical, and neurochemical abnormalities in aluminum chloride-induced Alzheimer’s disease rats. Biomed. Parmacother..

[83] Thancharoen O., Limwattananon C., Waleekhachonloet O., Rattanachotphanit T., Limwattananon P., Limpawattana P. (2019). *Ginkgo biloba* extract (EGb761), cholinesterase inhibitors, and memantine for the treatment of mild-to-moderate alzheimer’s disease: A network meta-analysis. Drugs Aging..

[84] Müller W.E., Eckert A., Eckert G.P., Fink H., Friedland K., Gauthier S., Hoerr R., Möller H.J., Ihl R., Kasper S. (2019). Therapeutic efficacy of the Ginkgo special extract EGb761^®^ within the framework of the mitochondrial cascade hypothesis of Alzheimer’s disease. The World J. Biol. Psychiatry.

[85] Yancheva S., Ihl R., Nikolova G., Panayotov P., Schlaefke S., Hoerr R. (2009). Ginkgo biloba extract EGb 761^®^, donepezil or both combined in the treatment of Alzheimer’s disease with neuropsychiatric features: A randomised, double-blind, exploratory trial. Aging Ment Health..

[86] Kehr J., Yoshitake S., Ijiri S., Koch E., Nöldner M., Yoshitake T. (2012). Ginkgo biloba leaf extract (EGb 761^®^) and its specific acylated flavonol constituents increase dopamine and acetylcholine levels in the rat medial prefrontal cortex: Possible implications for the cognitive enhancing properties of EGb 761^®^. Int. Psychogeriatr..

[87] Bastianetto S., Ramassamy C., Doré S., Christen Y., Poirier J., Quirion R. (2000). The ginkgo biloba extract (EGb 761) protects hippocampal neurons against cell death induced by β-amyloid. Eur. Neurosci..

[88] Nikmahzar E., Jahanshahi M., Babakordi F. (2018). *Ginkgo biloba* Extract Decreases Scopolamine-Induced Congophilic Amyloid Plaques Accumulation in Male Rat’s Brain. Jundishapur J. Nat. Pharm. Prod..

[89] Soheili M., Tavirani M.R., Salami M. (2012). Clearance of amyloid beta plaques from brain of Alzheimeric rats by *Lavandula angustifolia*. Neurosci Medic..

[90] Taiwo A.E., Leite F.B., Lucena G.M., Barros M., Silveira D., Silva M.V., Ferreira V.M. (2012). Anxiolytic and antidepressant-like effects of *Melissa officinalis* (lemon balm) extract in rats: Influence of administration and gender. Indian J. Pharmacol..

[91] Bounihi A., Hajjaj G., Alnamer R., Cherrah Y., Zellou A. (2013). In vivo potential anti-inflammatory activity of *Melissa officinalis* L. essential oil. Adv. Pharmcol. Sci..

[92] Leśniewicz A., Jaworska K., Żyrnicki W. (2006). Macro-and micro-nutrients and their bioavailability in polish herbal medicament. Food Chem..

[93] Hassanzadeh G., Pasbakhsh P., Akbar M., Shorki S., Ghahremani M., Amin G., Kashani I., Tameh A.A. (2013). Neuroprotective properties of *Melissa officinalis* L. extract against ecstasy-induced neurotoxicity. Cell J..

[94] Hu Y.R., Xing S.L., Chen C., Shen D.Z., Chen J.L. (2019). Tiaoxin Recipe, a Chinese herbal formula, inhibits microRNA-34a expression in the APPswe/PS1ΔE9 mouse model of Alzheimer’s disease. J. Integr. Med..

[95] Perry N.S., Houghton P.J., Sampson J., Theobald A.E., Hart S., Lis-Balchin M., Hoult J.R., Evans P., Jenner P., Milligan S. (2001). In-vitro activity of *S. lavandulaefolia* (Spanish sage) relevant to treatment of Alzheimer’s disease. J. Pharm. Pharmacol..

[96] Jukic M., Politeo O., Maksimovic M., Milos M., Milos M. (2007). In vitro acetylcholinesterase inhibitory properties of thymol, carvacrol and their derivatives thymoquinone and thymohydroquinone. Phytother. Res..

[97] Ayaz M., Sadiq A., Junaid M., Ullah F., Subhan F., Ahmed J. (2017). Neuroprotective and anti-aging potentials of essential oils from aromatic and medicinal plants. Front. Aging Neurosci..

[98] Eskandari-Roozbahani N., Shomali T., Taherianfard M. (2019). Neuroprotective Effect of Zataria Multiflora Essential Oil in Rats with Alzheimer’s Disease: A Mechanistic Study. Basic Clin. Neurosci..

[99] Suryawanshi M.V., Gujarathi P.P., Mulla T., Bagban I. (2024). Hypericum perforatum: A comprehensive review on pharmacognosy, preclinical studies, putative molecular mechanism, and clinical studies in neurodegenerative diseases. Naunyn-Schmiedeberg’s Arch. Pharmacol..

